# Effects of visual spatial frequency on audiovisual interaction: an event-related potential study

**DOI:** 10.3389/fnins.2025.1599114

**Published:** 2025-05-19

**Authors:** Fengxia Wu, Yanna Ren, Tengfei Hao, Jingjing Yang, Qiong Wu, Jiajia Yang, Meng Wang

**Affiliations:** ^1^School of Artificial Intelligence, Changchun University of Science and Technology, Changchun, China; ^2^Department of Psychology, College of Humanities and Management, Guizhou University of Traditional Chinese Medicine, Guiyang, China; ^3^Department of Psychology, Suzhou University of Science and Technology, Suzhou, China; ^4^Cognitive Neuroscience Laboratory, Graduate School of Natural Science and Technology, Okayama University, Okayama, Japan

**Keywords:** spatial frequency, visual orientation discrimination, audiovisual integration, early sensory stage, late cognitive stage, event-related potentials

## Abstract

Spatial frequency is a fundamental characteristic of visual signals that modulates the audiovisual integration behavior, but the neural mechanisms underlying spatial frequency are not well established. In the present study, the high temporal resolution of event-related potentials was used to investigate how visual spatial frequency modulates audiovisual integration. A visual orientation discrimination task was used, and the spatial frequency of visual stimuli was manipulated under three conditions. Results showed that the influence of visual spatial frequency on audiovisual integration is a dynamic process. The earliest audiovisual integration occurred over the left temporal-occipital regions in the early sensory stage (60–90 ms) for high spatial frequency conditions but was absent for low and middle spatial frequency conditions. In addition, audiovisual integration over fronto-central regions was delayed as spatial frequency increased (from 230–260 ms to 260–320 ms). The integration effect was also observed over parietal and occipital regions at 350–380 ms, and its strength gradually decreased at higher spatial frequencies. These discrepancies in the temporal and spatial distributions of audiovisual integration imply that the role of spatial frequency varies between early sensory and late cognitive stages. The findings of this study offer the first neural demonstration that spatial frequency modulates audiovisual integration, thus providing a basis for studying complex multisensory integration, especially in semantic and emotional domains.

## 1 Introduction

In our daily life, the brain continuously receives and processes sensory inputs from multiple modalities, such as vision, sound, touch, and smell. In particular, when auditory and visual signals are spatiotemporally congruent, the brain integrates them into unified perceptual representations to optimize behavioral responses—a phenomenon termed “audiovisual integration.” Previous studies have shown audiovisual integration across diverse domains, including spatial localization (Frassinetti et al., 2002), temporal judgment (Adams, 2016; Takeshima, 2024), object identification (Amedi et al., 2005; Van der Burg et al., 2011), and speech perception (Micheli et al., 2020; Stevenson and James, 2009). Audiovisual integration confers behavioral advantages such as faster response times (RTs) and higher accuracy for bimodal stimuli than for unimodal stimuli presented in isolation.

Audiovisual integration strongly depends on stimulus features such as the intensity and frequency of auditory stimuli (Green et al., 2019; Senkowski et al., 2011; Yang et al., 2015). Visual signal has two basic features: contrast and spatial frequency (SF). Previous studies have shown that a lower visual contrast leads to more audiovisual interactions than a higher visual contrast (Noesselt et al., 2010). Moreover, event-related potential (ERP) studies have shown that audiovisual integration is elicited by lower-contrast stimuli but not by higher-contrast stimuli 40–60 ms after stimulus presentation when visual and auditory stimuli are presented simultaneously (Senkowski et al., 2011). SF is an important characteristic of the visual system. Previous behavioral studies have established that contrast sensitivity across SFs in humans follows a characteristic inverted-U-shaped profile, showing maximum sensitivity to midrange frequencies, which decreases toward higher and lower frequencies (Campbell and Robson, 1968). The multichannel theory proposes that the visual system employs multiple independent, parallel channels to process visual information. Each channel is selectively attuned to a specific SF range—high frequencies for fine details (e.g., texture) and low frequencies for global structures (e.g., shape)—allowing for efficient hierarchical analysis of complex scenes. Indeed, some studies have shown that the primary visual cortex selectively processes visual-information-based visual SFs in both animals (De Valois et al., 1982; Issa et al., 2000) and humans (Sara and Sam, 2018). Furthermore, studies have been carried out on the human brain to investigate the neural mechanisms underlying the processing of changes in visual SFs (Baas et al., 2002; Musselwhite and Jeffreys, 1985). For example, an ERP study has revealed that the latency of the C1 component became longer with an increase in the SF (Musselwhite and Jeffreys, 1985). However, little is known about the interactions between visual stimuli of different SFs and auditory stimuli.

Several behavioral studies have investigated the influence of visual SF on audiovisual processing (Green et al., 2019; Jaekl and Soto-Faraco, 2010; Pérez-Bellido et al., 2013; Takeshima, 2024). In a visual orientation discrimination task, Jaekl and Soto-Faraco (2010) showed that auditory input selectively enhances contrast sensitivity at low SFs, as evidenced by significantly lower contrast thresholds in audiovisual stimuli than in visual-only stimuli. This integration effect of low SFs was further confirmed by Pérez-Bellido et al. (2013). Pérez-Bellido et al. (2013) instructed participants to detect visual speed to distinguish audiovisual enhancement due to stimulus-driven changes from those accounted for by decision-level contributions (Pérez-Bellido et al., 2013). Their results revealed that sound-induced visual enhancement was more selective for low-SF stimuli than for high-SF stimuli. Furthermore, Green et al. (2019) used an audiovisual simultaneity judgment task designed with different audiovisual stimulus onset asynchronies to investigate the role of visual SF in audiovisual integration (Green et al., 2019). Their results indicated that the temporal window of integration inside the human sensitivity range (1–12 c/d) was wider than that outside the human sensitivity range (Green et al., 2019). In a recent audiovisual simultaneity judgment study, Takeshima (2024) manipulated SF to probe its impact on temporal recalibration and revealed no significant differences in recalibration magnitude between low-SF (1.0 c/d) and high-SF (5.0 c/d) conditions (Takeshima, 2024). These behavioral studies demonstrate that visual SF differentially modulates audiovisual integration depending on task demands. However, there is little research on the role of SFs in the neural processing of audiovisual integration, and integration effects are yet to be investigated in detail.

In the present study, the neural mechanism underlying the effect of SFs on audiovisual integration was examined via the high temporal resolution of electroencephalogram (EEG). For this purpose, a visual orientation discrimination task was designed, and the SF of visual stimuli was manipulated under three conditions, namely low, middle, and high SFs. The nature and timing of audiovisual integration was analyzed by comparing the ERPs elicited by the audiovisual stimuli with the sum of the ERPs elicited by the unimodal auditory and unimodal visual stimuli. By comparing the differences in audiovisual integration among the three SFs, fundamental patterns regarding the influence of SF on audiovisual integration were identified: different stages and different SF modulation effects occur.

## 2 Materials and methods

### 2.1 Participants

Sixteen healthy volunteers (aged 22–29 years, mean age 24.1 years) from Okayama University participated in this experiment. All participants had normal or corrected-to-normal vision and were right-handed, with no neurological or psychiatric disorders and no hearing problems. The experimental protocol was approved by the Ethics Committee of Okayama University.

### 2.2 Stimuli and task

**Figure 1:**
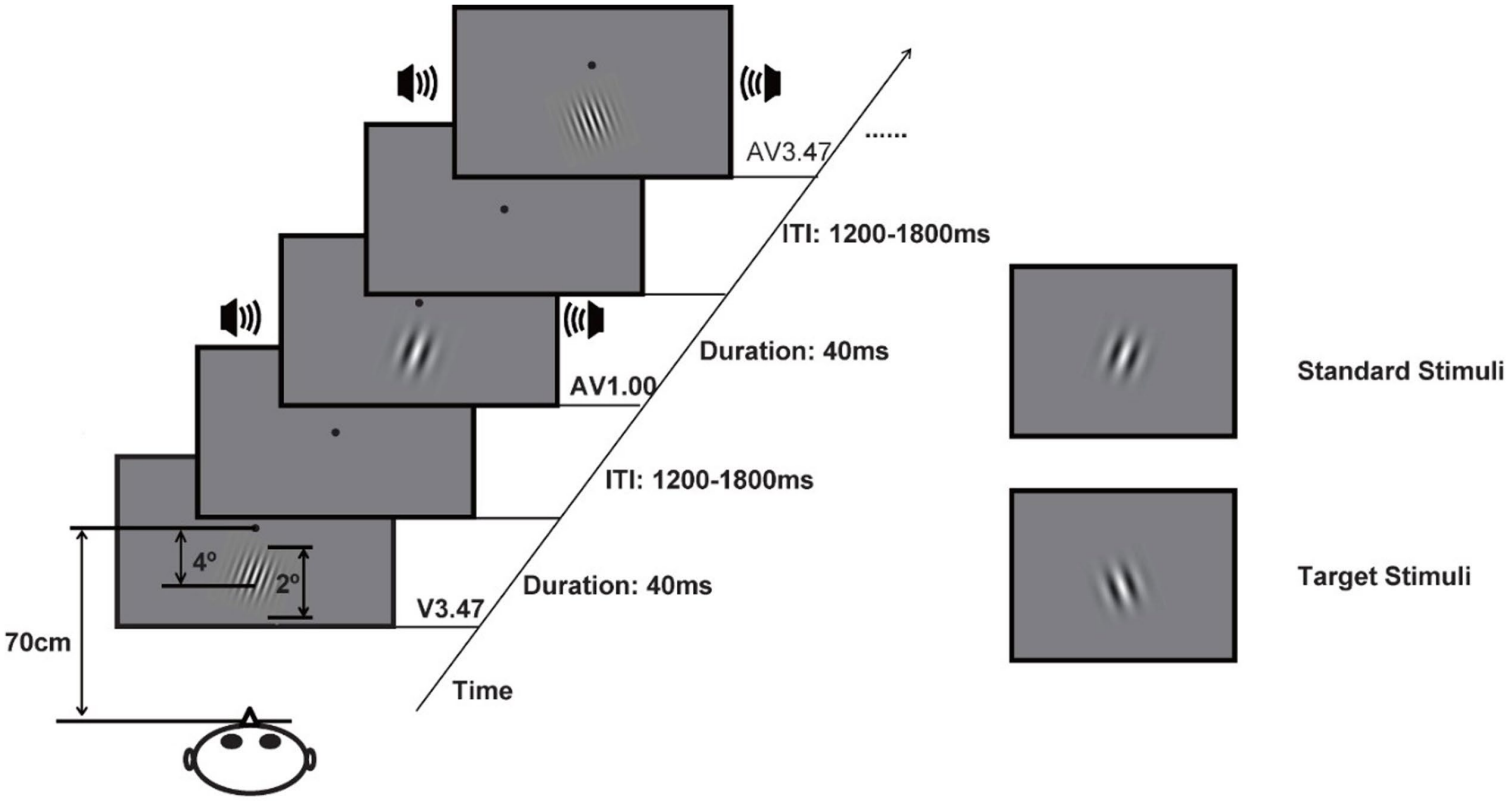
Experimental design. Stimuli were presented in a random stream of auditory stimuli, visual stimuli at three different frequencies, and audiovisual stimuli.

Stimuli presentation and response collection were carried out using MATLAB Release 14 with the Psychophysics Toolbox (PTB-3, a free, open-source collection of MATLAB and GNU Octave functions designed for designing and executing experiments in psychology research) (Brainard and David, 1997). The experiment consisted of three stimulus types: unimodal visual, unimodal auditory, and bimodal audiovisual (auditory and visual stimuli were presented simultaneously). The visual (V) stimuli consisted of a Gabor grating (2° visual angle, 30% contrast) with three SFs: 1.00 c/d, 1.86 c/d, and 3.47 c/d. These three SFs were selected based on previous studies (Campbell and Robson, 1968). The visual stimuli were presented on a 17-in. CRT monitor (100 Hz, 1,280 × 1,024 pixels, with a background luminance of 10 cd/m^2^) approximately 4° below the fixation point and included two subtypes with different orientations: clockwise 10° and anticlockwise 10°. The auditory stimulus (A) was a 3,000 Hz pure tone (65 dB SPL, 40 ms in duration, 5 ms rise and fall periods) that was presented through earphones. Bimodal audiovisual (AV) stimuli were presented at three levels, with the visual stimuli of 1.00 c/d (AV1.00), 1.86 c/d (AV1.86), and 3.47 c/d (AV3.47), as shown in Figure 1.

The experiment was performed in a dimly lit, electrically shielded, and sound-attenuated room (laboratory room, Okayama University, Japan). Each subject participated in 12 blocks, with each block lasting approximately 6 min. In six blocks, 10° clockwise was defined as the target visual stimulus, and 10° anticlockwise was defined as the standard reverse stimulus in the other six blocks. The order of orientation for the target stimulus was balanced between participants. Each block consisted of 54 visual stimuli (3 SFs × 15 standards and 3 SFs × 3 targets), 54 audiovisual stimuli (3 SFs × 15 standards and 3 SFs × 3 targets), 15 auditory stimuli, and 15 catch trials. The interstimulus interval varied randomly between 800 and 1,200 ms. At the beginning of each block, the participants were presented with a fixation point for 3,000 ms, and then, the stimuli were presented randomly for 40 ms. The participants were instructed to press the button as accurately and quickly as possible when the target stimuli were presented regardless of whether an auditory stimulus was presented, as shown in Figure 1.

### 2.3 Apparatus

An EEG system (BrainAmp MR plus, Gilching, Germany) was used to record EEG signals through 32 electrodes mounted on an electrode cap (Easy Cap, Herrsching-Breitbrunn, Germany). Horizontal eye movements were measured by deriving the electrooculogram (EOG) from one electrode placed approximately 1 cm from the outer canthi of the left eye. Vertical eye movements and eye blinks were detected by deriving an EOG from an electrode placed approximately 1.5 cm below the participant’s left eye. All signals were referenced to the left and right earlobes, and the impedance was maintained below 5 kΩ. Raw signals were acquired at a sample rate of 500 Hz and stored for offline analysis.

### 2.4 Behavioral data analysis

Hit rates, false alarms, and RTs were computed separately for each participant. RTs were calculated based on the responses falling within the mean RT ± 2.5 standard deviations (SDs). Hit rate was calculated as the percentage of correct responses relative to the total number of target stimuli, and false alarm rate was calculated as the percentage of incorrect responses relative to the total number of standard stimuli. In addition, perceptual sensitivity (*d’* = z(HR)-z(FA)) and response bias (*c* = −0.5 × [z(HR) + z(FA)]) were calculated for each participant. These measures were then analyzed using a 3 SF (1.00, 1.86, and 3.47 c/d) * 2 stimuli type (V and AV) repeated-measures ANOVA, followed by post-hoc tests with Bonferroni adjustment.

### 2.5 ERP data analysis

The EEG signals elicited by the standard stimuli were analyzed using BrainVision Analyzer software (version 1.0, Brain Products GmbH, Munich, Germany). First, the data were re-referenced to the average of left and right mastoids and bandpass-filtered from 1 to 30 Hz at a sample rate of 500 Hz. Next, the data were divided into epochs from −100 to 600 ms after stimulus onset, and baseline corrections were made from −100 to 0 ms. Then, epochs with a voltage exceeding ± 100 μV at any electrode location were excluded from the analysis. In addition, responses associated with false alarms were rejected. Finally, grand-averaged ERPs were obtained across all participants for each stimulus type. Three participants were excluded from further analysis due to the loss of more than 70% of the epochs over at least one stimulus type.

**Figure 2:**
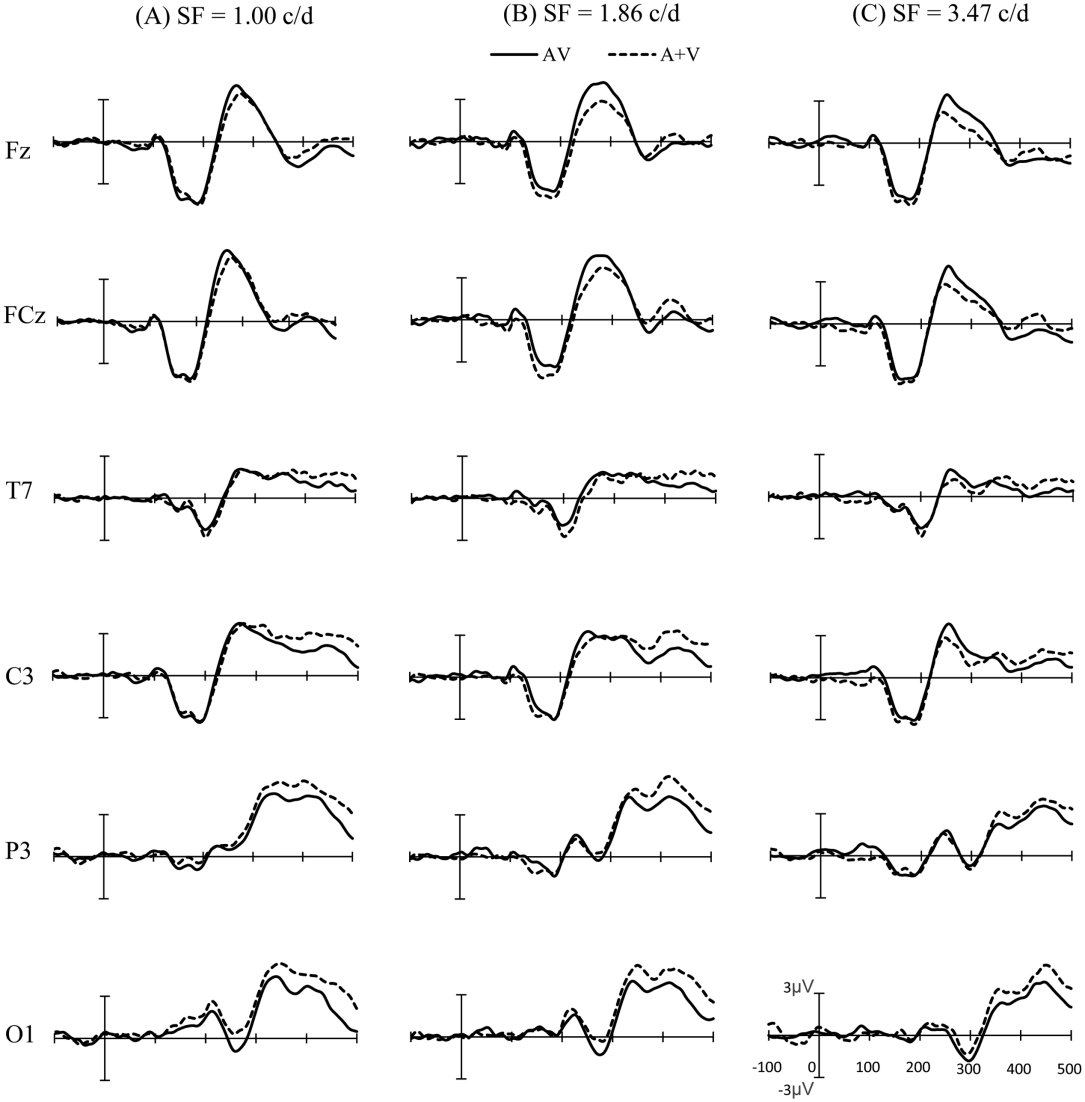
Event-related potentials of the sum of the unimodal stimuli (A+V) and bimodal (AV) stimuli at a subset of electrodes are shown from 100 ms before the stimulus to 500 ms after stimulus onset. **(A)** For the 1.00 c/d condition. **(B)** For the 1.86 c/d condition. **(C)** For the 3.47 c/d condition.

**Figure 3:**
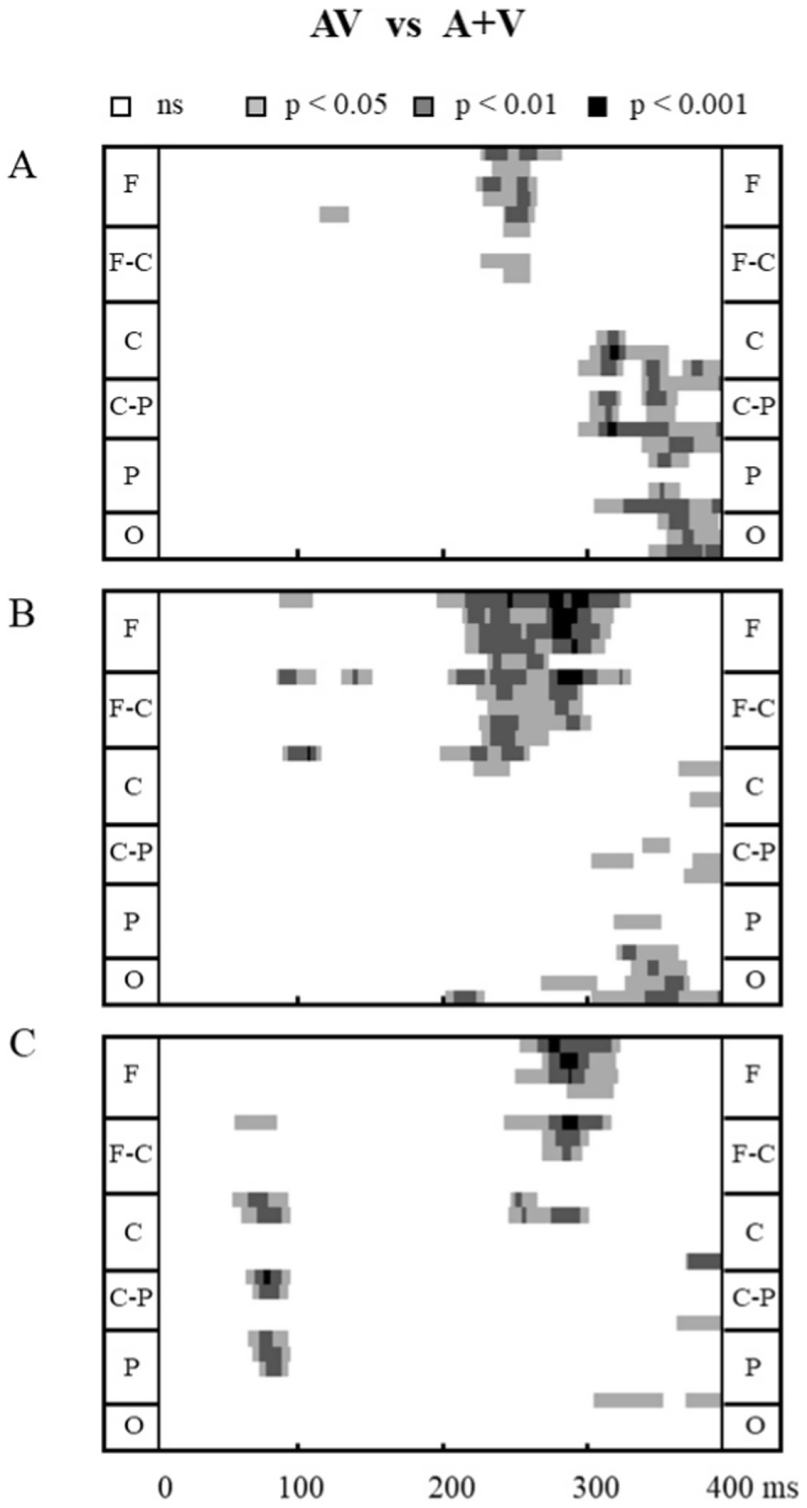
Statistical significance of audiovisual integration over all electrodes. The effects from point-wise running *t*-tests comparing AV with (A+V) for all participants when SF is 1.00 c/d **(A)**, 1.86 c/d **(B)**, and 3.47 c/d **(C)**. Time is plotted on the *x*-axis from 0 ms to 400 ms. Electrodes are plotted on the *y*-axis. Within a section, the electrodes are arranged from the left lateral to the right lateral sites. F, frontal; F-C, fronto-central; C, central; C-P, central-parietal; P, parietal; O, occipital.

To establish the presence of audiovisual integration, statistical analysis was conducted in three steps, following previous studies (Ren et al., 2018; Senkowski et al., 2011). First, the ERPs for bimodal AV stimuli were compared with the linear summation of unimodal auditory and unimodal visual ERPs (A+V) via pointwise running *t*-tests (two-tailed) for each electrode under each condition (1.00, 1.86, and 3.47 c/d). Significant differences were plotted when at least 12 consecutive data points met the alpha criterion of being < 0.05 (24 ms at a 500 Hz digitization rate was defined as audiovisual integration, Figure 2; Ren et al., 2018; Yang et al., 2015). Then, the regions of interest (ROI) and integration time intervals where and when significant audiovisual integration occurred were chosen based on the statistical analysis and topographical response patterns (Figure 3). In the second level of analysis, repeated-measures ANOVA were conducted for the three SFs of visual stimuli for the time interval and were selected based on an overview of the significant differences observed in the first step. Mean amplitude data were analyzed while accounting for the between-subject factors of stimulus types, conditions, electrodes, and time intervals. If a significant interaction between stimulus type, condition, or electrode and time interval was observed, the third phase of the analysis was carried out. In the third step, ANOVA were conducted separately for each of the ROI using the factor stimulus type (AV, A+V).

## 3 Results

### 3.1 Behavioral results

The RTs, hit rates, false alarm rates, perceptual sensitivity (*d’*), and response bias (c) are shown in Table 1. The 3 SF (1.00, 1.86, and 3.47 c/d) * 2 stimuli type (V and AV) repeated-measures ANOVA on RTs showed a main effect of stimuli type [*F* (1, 15) = 22.99, *p* = 0.000, η_p_^2^ = 0.605], indicating a faster response for AV stimuli than for V stimuli. In addition, a significant interaction was observed between stimuli type and SF [*F* (2, 30) = 8.86, *p* = 0.001, η_p_^2^ = 0.371]. Post-hoc comparisons showed significantly faster responses for AV versus V stimuli at SF 1.00 c/d (*p* < 0.01) and SF 1.86 c/d (*p* < 0.001), but not at SF 3.47 c/d. The 3 SF (1.00, 1.86, and 3.47 c/d) * 2 stimuli type (V and AV) repeated-measures ANOVA on hit rates revealed a significant main effect of SF [*F* (2, 30) = 13.65, *p* = 0.002, η_p_^2^ = 0.477], with a higher accuracy for SF 1.00 c/d (*p* = 0.005) and SF 1.86 c/d (*p* = 0.006) than for SF 3.47 c/d. Furthermore, a main effect of stimuli type was observed [*F* (1, 15) = 9.05, *p* = 0.009, η_p_^2^ = 0.376], where AV stimuli elicited larger hit rates than V stimuli. This auditory enhancement was significant only for SF 1.86 c/d (*p* = 0.049) and SF 3.47 c/d (*p* = 0.037). However, the interaction between SF and stimuli type was not significant [*F* (2, 30) = 1.55, *p* = 0.233, η_p_^2^ = 0.094]. The 3 SF (1.00, 1.86, and 3.47 c/d) * 2 stimuli type (V and AV) repeated-measures ANOVA on false alarm rates revealed a significant main effect of stimuli type [*F* (1, 15) = 7.39, *p* = 0.016, η_p_^2^ = 0.330], with higher false alarms for AV stimuli than for V stimuli. The 3 SF (1.00, 1.86, and 3.47 c/d) * 2 stimuli type (V and AV) repeated-measures ANOVA on *d’* showed a main effect of SF [*F* (2, 30) = 4.58, *p* = 0.035, η_p_^2^ = 0.234], indicating decreased sensitivity with increasing SF. No significant interaction was observed [*F* (2, 30) = 1.78, *p* = 0.192, η_p_^2^ = 0.106] though a marginal difference between V and AV stimuli emerged at SF 3.47 c/d (*p* = 0.062). The 3 SF (1.00, 1.86, and 3.47 c/d) * 2 stimuli type (V and AV) repeated-measures ANOVA on *c* showed a main effect of SF [*F* (2, 30) = 9.16, *p* = 0.004, η_p_^2^ = 0.379] and a main effect of stimuli type [*F* (1, 15) = 11.29, *p* = 0.004, η_p_^2^ = 0.429], but no interaction was observed.

**Table 1:** Mean behavioral data for all participants in the experiment.

Stimulus types	Response times (ms)	Hit rates (%)	False alarm rates (%)	*d’*	*c*
V1.00	533.6 ± 14.52	95.3 ± 1.14	0.71 ± 0.14	4.31 ± 0.15	0.36 ± 0.07
V1.86	542.9 ± 16.41	92.6 ± 1.86	1.04 ± 0.26	4.01 ± 0.17	0.39 ± 0.08
V3.47	581.6 ± 18.93	78.9 ± 4.61	0.67 ± 0.23	3.53 ± 0.18	0.76 ± 0.12
AV1.00	508.1 ± 15.77	95.6 ± 1.44	1.04 ± 0.22	4.29 ± 0.15	0.29 ± 0.08
AV1.86	497.1 ± 11.51	95.0 ± 1.21	1.47 ± 0.36	4.05 ± 0.14	0.26 ± 0.07
AV3.47	580.1 ± 17.73	84.8 ± 3.67	0.66 ± 0.22	3.73 ± 0.18	0.65 ± 0.09

Data are presented as the mean ± standard error of the mean (SEM). V, visual stimulus; AV, audiovisual stimulus.

### 3.2 ERP results

**Figure 4:**
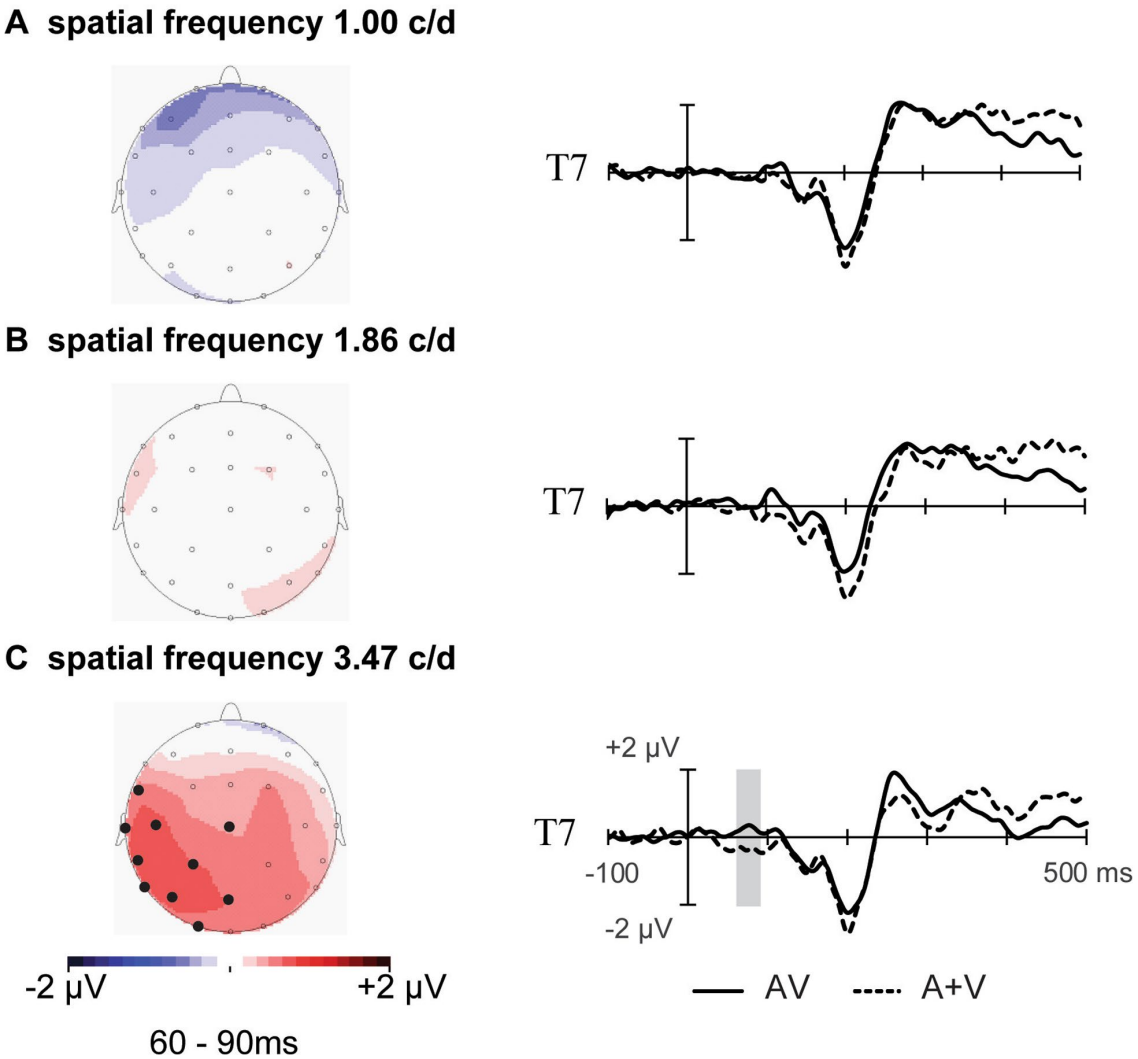
Topography of the significant spatiotemporal patterns of integration in the left temporal-occipital regions. Right sides: event-related potentials of the sum of the AV and A+V stimuli at electrode T7 are shown from 100 ms before the stimulus to 500 ms after the stimulus. The shaded areas indicate the time periods when the AV response significantly differs from the A+V responses (*p* < 0.05).

The average ERPs across the three experimental conditions are shown in Figure 2. The results of pointwise running *t*-tests comparing AV and A+V conditions at the three different SFs are shown in Figure 3. Based on the statistical analysis and topographical response patterns, six ROI (frontal: F7, F3, Fz, F4, F8; fronto-central: FC5, FC1, FCz, FC2, FC6; central: C3, Cz, C4; parietal: P3, Pz, P4; occipital: O1, Oz, O2; and temporal: T7, T8) and three integration time windows (60–90 ms, 230–320 ms, and 350–380 ms) were selected. Due to significant lateralization effects in the early time interval of 60–90 ms (Figure 4 topography), bilateral electrodes within each ROI were selected for subsequent analyses (left: F3, FC1, T7, C3, P3 and O1; right: F4, FC2, T8, C4, P4 and O2). A 3 SF (1.00, 1.86, and 3.47 c/d) * 2 stimuli type (AV and A+V) * 3 time interval (60–90 ms, 230–320 ms, and 350–380 ms) * 6 ROI (frontal, fronto-central, temporal, central, parietal, and occipital) * 2 electrode (left and right) repeated-measures ANOVA on mean amplitudes revealed a significant five-way interaction [*F* (20, 240) = 2.64, *p* = 0.016, η_p_^2^ = 0.180]. This indicates the distinct audiovisual integration patterns for different SF conditions in different time intervals. Therefore, these differences were analyzed in detail in the following sections.

#### 3.2.1 Audiovisual integration for 60–90 ms

In the early stage of 60–90 ms, audiovisual integration occurred for SF 3.47 c/d, but not for SF 1.00 and SF 1.86 c/d (Figures 2, 4). A 2 stimuli type (AV and A+V) * 6 ROI (frontal, fronto-central, temporal, central, parietal, and occipital) * 2 electrode (left and right) repeated-measures ANOVA was conducted for SF 3.47 c/d. A significant main effect of stimuli type was observed [*F* (1, 12) = 13.04, *p* = 0.004, η_p_^2^ = 0.521], with more positive amplitudes for the ERPs elicited by AV than those elicited by A+V. In addition, a significant interaction between stimuli type* ROI *electrode was observed [*F* (5, 60) = 4.48, *p* = 0.008, η_p_^2^ = 0.272]. Post-hoc analyses revealed a significant difference at electrodes FC1, T7, C3, P3, P4, and O1 (all *p* < 0.05). These results indicate that significant early audiovisual integration occurred for high-SF stimulus over left temporal-occipital regions, as shown in Figure 4C.

#### 3.2.2 Audiovisual integration for 230–320 ms

**Figure 5:**
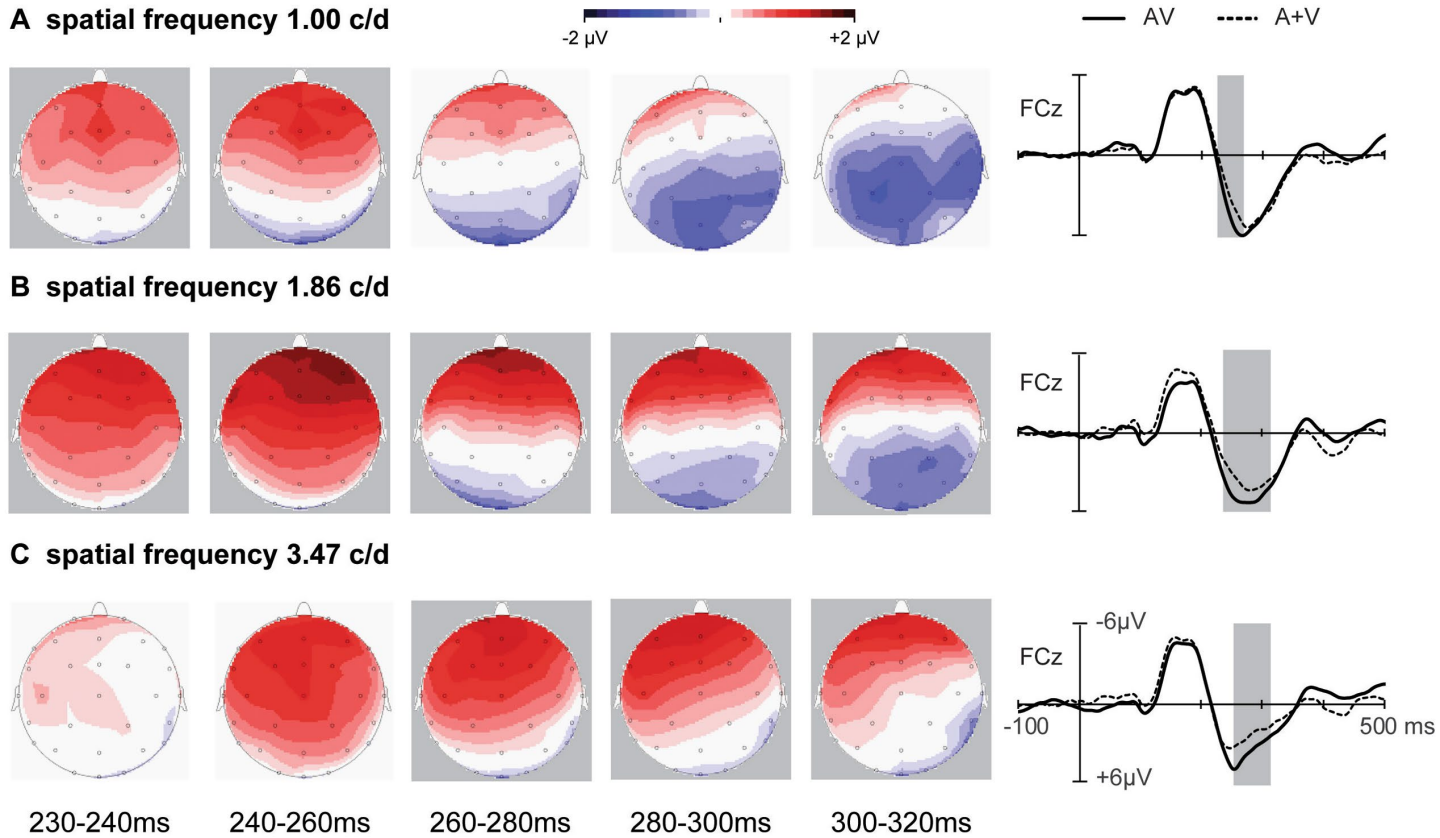
Topography of the different significant spatiotemporal patterns of integration in the fronto-central regions. Right sides: event-related potentials of the sum of the AV and A+V stimuli at electrode FCz are shown from 100 ms before the stimulus to 500 ms after the stimulus. The shaded areas indicate the time periods when the AV response significantly differs from the A+V responses (*p* < 0.05).

The ERPs and topography maps within the 230–320 ms window are shown in Figure 5. The onset time and duration of audiovisual integration differed significantly across the three SF conditions in frontal and fronto-central regions. Separate three-way ANOVA (2 stimuli type (AV and A+V) * 6 ROI (frontal, fronto-central, temporal, central, parietal, and occipital) * 2 electrode (left and right)) was carried out for each condition using average ERP amplitudes. For SF 1.00 c/d, audiovisual integration occurred within 230–260 ms. ANOVA revealed a significant main effect of ROI [*F* (5, 60) = 7.40, *p* = 0.009, η_p_^2^ = 0.381] and a significant ROI * stimuli type interaction [*F* (5, 60) = 7.80, *p* = 0.003, η_p_^2^ = 0.374]. Post-hoc analyses showed a larger amplitude for AV than for A+V in frontal (*p* = 0.004) and fronto-central (*p* = 0.027) regions. For SF 1.86 c/d, audiovisual integration was observed in 230–320 ms, with a significant main effect of ROI [*F* (5, 60) = 7.44, *p* = 0.010, η_p_^2^ = 0.383] and a significant ROI * stimuli type interaction [*F* (5, 60) = 11.32, *p* = 0.001, η_p_^2^ = 0.485]. Post-hoc tests indicated significant audiovisual integration in frontal (*p* = 0.003), fronto-central (*p* = 0.006), and temporal regions (*p* = 0.015). For SF 3.47 c/d, audiovisual integration occurred later, within 260–320 ms, showing a significant main effect of ROI [*F* (5, 60) = 14.12, *p* = 0.001, η_p_^2^ = 0.541] and a significant ROI*stimuli type interaction [*F* (5, 60) = 9.20, *p* = 0.001, η_p_^2^ = 0.434]. Post-hoc analyses confirmed audiovisual integration in frontal (*p* = 0.013) and fronto-central (*p* = 0.041) regions. This finding is of particular interest because it shows that the effects of higher SF stimuli on audiovisual integration processes can occur later.

#### 3.2.3 Audiovisual integration for 350–380 ms

**Figure 6:**
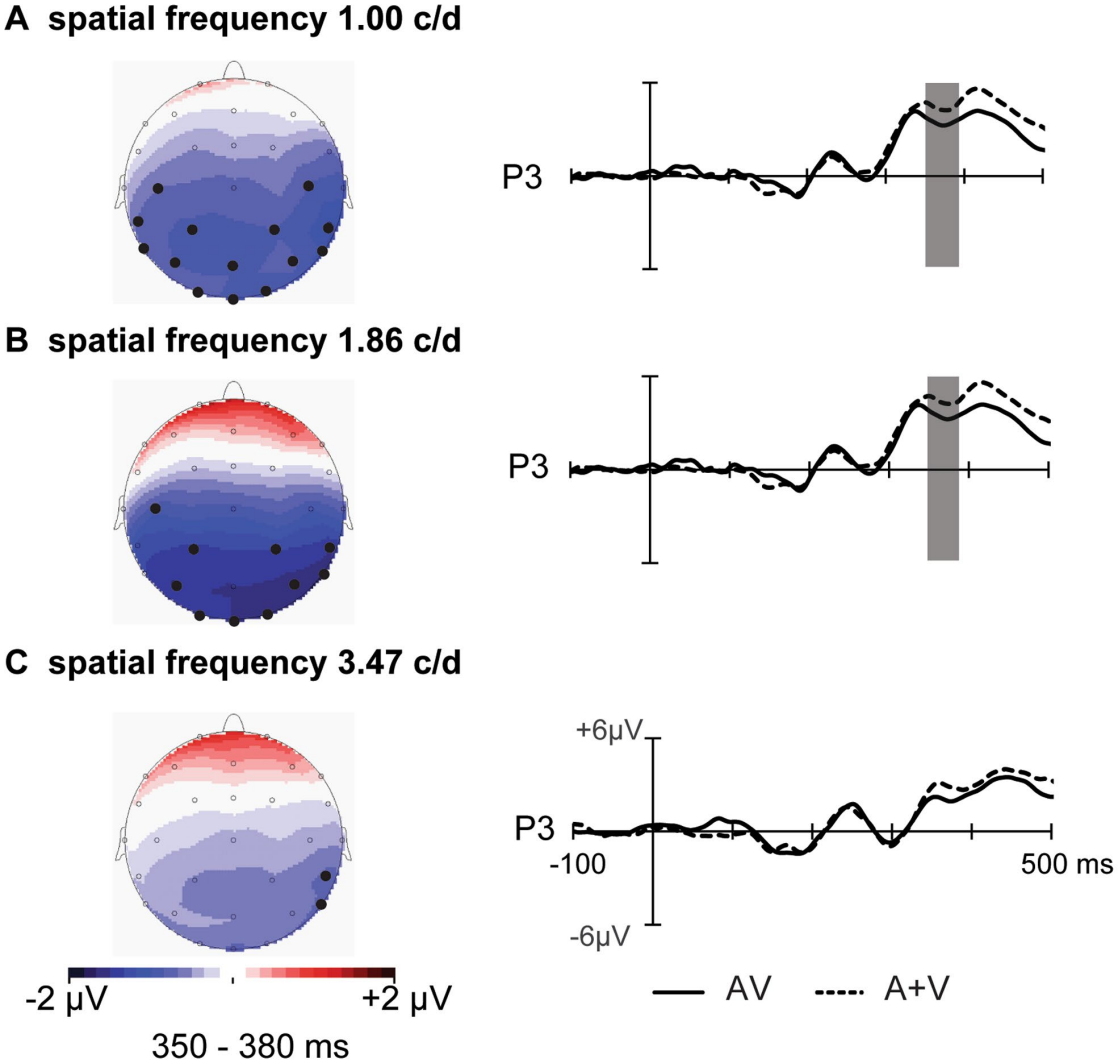
Topography of the different significant spatiotemporal patterns of integration in the parieto-occipital regions. Right sides: event-related potentials of the sum of the AV and A+V stimuli at electrode P3 are shown from 100 ms before the stimulus to 500 ms after the stimulus. The shaded areas indicate the time periods when the AV response significantly differs from the A+V responses (*p* < 0.05).

The ERPs and topography maps within the 350–380 ms window are shown in Figure 6. For SF 1.00 c/d, ANOVA revealed a significant main effect of stimuli type [*F* (1, 12) = 7.45, *p* = 0.018, η_p_^2^ = 0.383] and ROI [*F* (5, 60) = 12.86, *p* = 0.002, η_p_^2^ = 0.517]. Post-hoc comparisons showed significantly smaller amplitudes for AV than for A+V stimuli in central (*p* = 0.038), parietal (*p* = 0.004), and occipital (*p* = 0.005) regions. In addition, a significant stimuli type* ROI *electrode interaction was observed [*F* (5, 60) = 4.68, *p* = 0.004, η_p_^2^ = 0.281], with post-hoc tests revealing audiovisual integration at electrodes C3, P3, P4, O1, O2, and T8 (all *p* < 0.05). For SF 1.86 c/d, ANOVA revealed a significant main effect of ROI [*F* (5, 60) = 12.67, *p* = 0.001 = 2, η_p_^2^ = 0.513] and a significant ROI * electrode interaction [*F* (5, 60) = 3.27, *p* = 0.046, η_p_^2^ = 0.214]. Post-hoc analyses showed larger amplitudes in right electrodes than in left electrodes in parietal (3.69 μV vs. 5.13 μV, *p* = 0.050) and occipital (3.93 μV vs. 4.70 μV, *p* = 0.015) regions. Furthermore, a significant ROI * stimuli type interaction was also observed [*F* (5, 60) = 5.16, *p* = 0.011, η_p_^2^ = 0.301], with AV amplitudes smaller than A+V amplitudes in parietal (*p* = 0.039) and occipital (*p* = 0.011) regions. However, there was no significant main effect of stimuli type or stimuli type* ROI interaction for SF 3.47 c/d (all *p* > 0.05), with only a significant main effect of ROI [*F* (5, 60) = 12.98, *p* = 0.001, η_p_^2^ = 0.520] and ROI *electrode interaction [*F* (5, 60) = 4.11, *p* = 0.023, η_p_^2^ = 0.255]. Post-hoc tests confirmed larger amplitudes in right electrodes than in left electrodes in parietal (*p* = 0.019) and occipital (*p* = 0.009) regions. These results indicate that audiovisual integration over parieto-occipital regions is decreased with increasing visual SF.

## 4 Discussion

In the present study, we aimed to investigate the neural mechanism by which visual SF modulates audiovisual integration using a visual orientation discrimination task. Our results clearly revealed that SF influences audiovisual integration and that different patterns of SF modulation occur in the early perceptual and late cognitive stages.

### 4.1 SF modulates audiovisual integration in a visual orientation discrimination task

At the behavioral level, our results showed that responses to audiovisual stimuli were faster and more accurate than responses to visual stimuli, suggesting that the presence of a synchronous auditory stimulus facilitates visual orientation discrimination (Busse et al., 2005; Frassinetti et al., 2002; Li et al., 2015; Lippert et al., 2007). This facilitating effect on RTs significantly increased as the SF increased from 1.00 to 1.86 c/d, whereas it disappeared at 3.47 c/d. It seems that part of the attention necessary for cross-modal processing (Busse et al., 2005; Donohue et al., 2011) shifted to identify visual orientation as the SF increased, resulting in the disappearance of audiovisual facilitation. This explanation is consistent with sensitivity (*d’*) results, which decreased with increasing SF for both audiovisual and visual stimuli (1.00 > 1.86 > 3.47, all *p* < 0.05), suggesting that the participants’ orientation discrimination ability decreased as the SF increased from 1.00 to 3.47 c/d (Musselwhite and Jeffreys, 1985; Pérez-Bellido et al., 2013). However, the facilitating effect was significantly higher with increasing SF in *d’*. This observation is in line with those of previous studies (Bolognini et al., 2010; Corneil et al., 2002; Noesselt et al., 2010), which demonstrated an inverse relationship between audiovisual benefits and stimulus intensity. In addition, behavioral results revealed that there may be differences in the effects of SFs on audiovisual integration during different stages.

### 4.2 Audiovisual integration at the early sensory stage

As shown in Figures 2, 3, our results revealed that audiovisual integration occurred as early as 60–90 ms over temporal-occipital regions. This early latency was in line with previous studies that reported low-level sensory interactions, which are considered the earliest stage of multisensory processing within 100-ms post-stimulus onset, allowing the brain to automatically select and encode external inputs that can facilitate simultaneous stimulus encoding (Cappe et al., 2010; Giard and Peronnet, 1999; Meo et al., 2015; Molholm et al., 2002; Senkowski et al., 2011; Talsma et al., 2007; Van der Burg et al., 2011). Therefore, the task-irrelevant auditory stimulus in the present study can interact with the simultaneous visual stimulus during the early visual sensory processing stage, thereby enhancing the perceptual salience of the visual stimulus. Importantly, our results revealed a left lateralization effect over temporal-occipital regions. Some studies have reported ERPs similar to the left lateralization effect over posterior regions under visual or auditory selective attention (Starke et al., 2017; Van der Burg et al., 2011). For example, in the study by Van der Burg et al. (2011), participants were asked to perform a visual search task to investigate early audiovisual stimuli and were required to report the orientation of the target visual stimulus that was present or not present with the auditory stimulus (Van der Burg et al., 2011). Their results revealed early audiovisual integration at 50–60 ms over the left parieto-occipital region. Furthermore, Starke et al. (2017) used combined functional magnetic resonance imaging and EEG methods to investigate the neural basis of the visually induced enhancement of auditory detection via auditory detection tasks (Starke et al., 2017). They reported that visual-induced auditory enhancement involved left lateralization for low-intensity sounds over the superior temporal sulcus. However, Senkowski et al. (2011) reported that even if attention is directed to both modalities simultaneously, the left lateralization effect occurs during the earliest integration (Senkowski et al., 2011). Their results revealed an ERP component at 40–60 ms over the left posterior and right anterior regions for the low-intensity condition. Therefore, both attention and stimulus intensity may influence early audiovisual stimuli integration over occipital-temporal regions, and further electrophysiological studies are required to investigate this observation in detail.

Notably, the earliest audiovisual integration was found in the 3.47 c/d condition but was absent in the 1.00 and 1.86 c/d conditions (Figure 4). This phenomenon may be related to the intensity of the visual stimulus; a high-SF visual signal tends to be perceived as having a lower intensity than low-SF visual signals when contrast is constant (Georgeson and Sullivan, 1975). Our behavioral results confirmed this finding, showing that visual perceptual ability (*d’*) decreased with increasing SF from 1.00 c/d to 3.47 c/d (Table 1). Some studies have shown that stimulus intensity can modulate audiovisual integration (Fort et al., 2002; Senkowski et al., 2011; Stevenson and James, 2009). For instance, Fort et al. (2002) manipulated the orientation of visual stimuli (horizontal and vertical) and frequency of sounds (540 Hz and 560 Hz with a low intensity of 50 dB) to investigate the neural mechanism of audiovisual integration in simple detection tasks (Fort et al., 2002). Their results revealed that early integration occurred at 45–85 ms over the occipital-parietal regions when their participants were presented with a low-intensity auditory stimulus. Furthermore, Senkowski et al. (2011) manipulated the intensity of auditory, visual, and audiovisual stimuli of low, middle, and high levels to investigate the effects of stimulus intensity on multisensory audiovisual processing. Their results also revealed an early audiovisual interaction (40–60 ms), showing that the integration effect occurred particularly for low-intensity inputs but not for stimuli with middle and high intensities. Overall, in these studies, the earliest integration occurred when at least one of the presented input modalities (auditory and/or visual) was relatively low in stimulus intensity, which follows the principle of inverse effectiveness. Therefore, we speculated that a high-SF-evoked audiovisual integration at the early sensory stage was dependent on stimulus intensity. However, in the present study, only SFs ranging from 1.00 to 3.47 c/d were presented; thus, our study does not allow us to draw conclusions about how high an SF needs to be to evoke an early integration effect. Further electrophysiological studies are needed to elucidate the neural mechanisms of integration under more detailed visual SF conditions.

### 4.3 Audiovisual integration at the late cognitive stage

Audiovisual integration was significantly delayed with increased SFs over the frontal and fronto-central regions (Figure 5). Audiovisual integration was observed at 230–260 ms, 230–320 ms, and 260–320 ms at 1.00 c/d, 1.86 c/d, and 3.47 c/d, respectively. This phenomenon may be related to the two visual processing pathways of the dorsal and ventral streams (Fang and He, 2005; Haxby et al., 1991). A visual stimulus with low-SF information is primarily processed through the dorsal stream, which responds relatively faster, whereas high-SF information is projected chiefly to the ventral stream, with fine resolution but slow responses (Parker, 1980; Tootell and Nasr, 2017; Zhang et al., 2015). Consistent with our findings, previous behavioral investigations revealed that audiovisual integration occurs through both the dorsal and ventral pathways by processing low SFs (Jaekl and Soto-Faraco, 2010) and high SFs (Jaekl and Harris, 2009), respectively. Thus, audiovisual stimuli of 1.00 c/d allow the stimulus to reach high-order areas rapidly, primarily via the dorsal visual stream, and integrate with the auditory stimulus, whereas audiovisual stimuli of 3.47 c/d require more time to receive high-order areas, primarily via the ventral visual stream, and integrate with the auditory stimulus, resulting in delayed audiovisual integration. Importantly, no stimulus is processed exclusively through the ventral or the dorsal pathway. Both visual and auditory stimuli have been shown to be processed in parallel processing streams (Merigan and Maunsell, 1993; Nassi and Callaway, 2009). This parallel processing has subsequently been confirmed in audiovisual integration (Ahveninen et al., 2016; Kaposvári et al., 2015). Therefore, audiovisual stimuli of 1.86 c/d allow parallel processing in two pathways, leading to a wider integration time interval (230–320 ms), which combines 1.00 c/d with 3.47 c/d. Overall, we speculate that integration over frontal and fronto-central regions was influenced by the visual processing pathway and that a high SF delayed audiovisual integration.

In addition, our results revealed another instance of audiovisual integration at 350–380 ms over the parietal and occipital regions. Similar to our observations, audiovisual integration activity over the parietal and occipital regions has also been reported in some previous studies in which participants were asked to perform visual-direction-related tasks (Kayser et al., 2017; Yang et al., 2013). Yang et al. (2013), using the same visual orientation discrimination task to investigate the influence of sound location on audiovisual integration, reported significant integration in the parietal and occipital regions at 360–400 ms (Yang et al., 2013). More recently, Kayser et al. (2017) used the visual motion direction discrimination task and reported that sound facilitates visual motion discrimination by enhancing occipital visual representations at approximately 350 ms after stimulus onset. However, integration in these regions was absent for 3.47 c/d. This absence may be related to visual perceptual load, which has been elucidated to significantly influence audiovisual processing (De Niear et al., 2016; Macdonald and Lavie, 2011). Macdonald and Lavie (2011) reported that participants were less able to notice the presence of a simple auditory tone in the last trial while they were performing a high-visual-load task than when they were performing a low-visual-load task. Furthermore, Gibney et al. (2017) provided further evidence for the effect of visual perceptual load on audiovisual integration using audiovisual speed detection tasks via dual-task paradigms. Their results revealed that audiovisual integration occurred under no and low perceptual load conditions but was absent under high perceptual load conditions. Indeed, in the present study, discriminating visual orientation with under the 3.47 c/d condition was the most difficult task (Musselwhite and Jeffreys, 1985; Pérez-Bellido et al., 2013), and participants needed to pay more attention to this orientation, which was also necessary for cross-modal audiovisual processing to identify the orientation (Busse et al., 2005; Donohue et al., 2011), resulting in the absence of audiovisual integration. Specifically, our results revealed that the activity in the parietal and occipital regions was particularly relevant to the enhancement of behavioral performance (the absence of audiovisual benefits in RTs). It was previously noted that the activity over the parietal and occipital regions results in task-relevant representations. Therefore, we speculated that modulations in the parietal and occipital regions may be dependent on feedback from higher association areas, which guide audiovisual influences based on task requirements.

## 5 Conclusion

In summary, the present study investigated the neural mechanism by which visual SFs modulate audiovisual integration during a visual orientation discrimination task. Our results showed that the modulatory effect in the brain was a dynamic process. In the early sensory stage (60–90 ms), audiovisual integration occurred in temporal-occipital regions as the SF increased, which may reflect an automatic, bottom-up intersensory mechanism that can increase perception depending on stimulus intensity. In the late cognitive stage, audiovisual integration was delayed (230–320 ms) over fronto-central regions and attenuated (350–380 ms) over parieto-occipital regions with increasing SFs, which may reflect a top-down mechanism that is influenced by the signal processing pathway and task requirements. Taken together, our findings can be useful for further studies that investigate the integration of complex stimuli, especially emotional and semantic integration.

## Data Availability

The datasets and materials generated during the current study are available from the corresponding author upon reasonable request.
